# Burden of road traffic injuries and related risk factors in low and middle-income Pacific Island countries and territories: a systematic review of the scientific literature (TRIP 5)

**DOI:** 10.1186/1471-2458-12-479

**Published:** 2012-06-25

**Authors:** Josephine Herman, Shanthi Ameratunga, Rod Jackson

**Affiliations:** 1 Section of Epidemiology and Biostatistics, School of Population health, University of Auckland, Private Bag 92019, Auckland, New Zealand

## Abstract

**Background:**

In Pacific Island countries and territories, the burden of road traffic injuries and their attendant risks are considered significant but are poorly quantified. As with other low and middle-income countries, understanding the epidemiology of road traffic injuries in Pacific countries is critical to informing sustainable research and policy initiatives aimed at reducing this burden.

**Methods:**

We undertook a systematic review and critical appraisal of the relevant epidemiological literature between January 1980 and December 2010, using key search strings for incidence and aetiological studies focusing on RTIs in less resourced Pacific countries.

**Results:**

Nineteen studies were identified. The majority were descriptive and were unable to provide population-based estimates of the burden of road crash injury, or reliable information on risk factors using well-designed aetiological research methods. All studies were published more than 10 years ago, and all but three reported on data from Papua New Guinea, thereby limiting the generalisability of findings to the current status in the region. Studies undertaken in Papua New Guinea suggested that RTIs were more frequent among young males, with head injuries the most common cause of death or hospital admission. Two thirds of fatalities occurred at the crash site or soon after admission. Most road crash victims were passengers or pedestrians. Factors postulated to influence the risk of RTIs were travel in open-back utility vehicles, utility vehicle overcrowding, and alcohol.

**Conclusions:**

This review suggests that, despite increasing awareness of the importance of addressing road safety among stakeholders in less resourced Pacific Island countries, road traffic injuries have not been a research priority with little relevant current evidence from the region to inform policy. Robust epidemiological research that can assess the magnitude and key determinants of road traffic injuries in these settings is essential to determine context-specific road safety initiatives that are relevant and affordable. Greater attention to harnessing routinely collected data (e.g., hospital information systems and police crash statistics) to inform policy is also required.

## Background

Road traffic injuries (RTIs) are a significant public health challenge and projected to be the fifth leading contributor to the global burden of disease by 2030 [1]. Over 90% of fatal crashes occur in low and middle-income countries with substantial consequences, particularly for vulnerable road users such as pedestrians, cyclists, and riders of motorised two-wheelers [2-4]. In Pacific Island countries and territories, the published literature suggests that RTIs and their attendant risks are a significant but poorly quantified cause of death and disability [5-7]. It has been reported that up to half of all fatal injuries in the Pacific are due to road traffic crashes [8]. Previous commentators and researchers have also drawn attention to the impact of RTIs among Pacific populations, highlighting factors that require particular attention such as alcohol, motorisation, poor driving, seatbelt use, and poorly maintained vehicles and roads [9-15]. The Global road safety survey identifies Pacific Island country-specific characteristics from the perspectives of government statistics and stakeholders [16]. However in order to develop effective road safety initiatives that are relevant for the Pacific context, it is important to assess the quality of local research evidence examining the burden and risk factors for road crashes, and gain a broader appreciation of its utility to influence national policy and behavioural change [17-19]. To this end, we undertook a systematic review of the published literature of epidemiological studies investigating the burden of and modifiable risk factors for RTIs in less resourced Pacific countries.

## Methods

We identified and critically appraised epidemiological studies published between January 1980 and December 2010 focusing on RTIs. The countries of interest comprised 18 of the 22 member states of the Secretariat of the Pacific Community, encompassing all of the countries in the Pacific region but excluding high-income countries as classified by The World Bank [20]. Studies describing the burden of RTIs were included if they presented epidemiological data on road users who had sustained an injury as a result of a road traffic crash. Studies investigating risk factors for RTI were reviewed if they included a comparison group or time period (as in case crossover studies) with respect to the exposure of interest, and the study outcome measure was a road traffic crash resulting in an injury (fatal or non-fatal). Therefore the primary study designs of relevance included: case control, cohort, case crossover, or cross sectional research methodologies. The following electronic databases were searched building on strategies recommended for reviewing the literature addressing incidence and aetiological studies [21]: Medline, EMBASE, CINAHL, PsycINFO and the Australian Transport and Road Index database, and Transport Research International Documentation. A search string applied for the review is provided in additional material. (Additional file 1). The keywords and relevant medical subject heading terms used included: specific names of all eligible Pacific Island countries and territories, Polynesia, Melanesia, Micronesia, Oceania, road user, driver, occupant, passenger, pedestrian, motor, vehicle, cycle, bike, moped, automobile, car, walking, traffic, road, accident, crash, collision, casualty, trauma, wound, injury, fatal, death, disability. Specific search terms for risk factors included alcohol, wine, spirit, beer, seatbelt, helmet, head protective device, sleep, fatigue, apnoea, conspicuity, visibility, illumination, visual, headlights, light, colour, contrast. Further studies, reports, conference proceedings, and other relevant publications in the grey literature were located through the reference lists of identified articles and websites of groups involved in injury and Pacific health research. In addition, we undertook hand and electronic searches of the PNG MED J (1972–2009), Fiji Medical Journal (1974 - October 2006), and Pacific Health Dialogue (1994–2010), as well as journals with a focus on injury, specifically Accident Analysis and Prevention, Injury, Injury prevention, Journal of Injury Control and Safety Promotion, Journal of Safety Research, and Traffic Injury Prevention.

**Figure 1:**
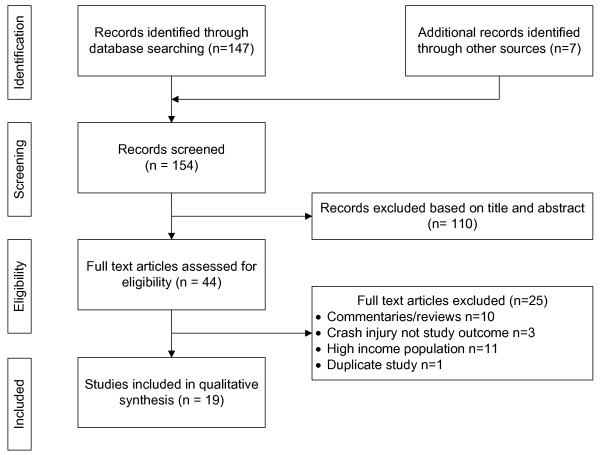
Flow diagram of studies selected for review.

The review was restricted to studies published in the English language. All studies identified were screened initially by title and abstract, and then more thoroughly in full text. (Figure 1) Adhering to PRISMA guidelines,1 (Additional file 2) studies meeting the inclusion criteria were critically appraised for the quality of the evidence using GATE-LITE™ [22] and summarised in evidence tables with the following headings: study design, participants, variables examined, key findings relevant to the review and study appraisal and comments. (Table 1).

**Table 1:** Study descriptions

**Study design**	**Participants**	**Variables examined**	**Key findings relevant to review**	**Study appraisal/Comments**
1. Wyatt [23] (1980), PNG, Case series Retrospective	121 RTI-related forensic autopsies PMGH, 1975-78.	Road user type, injury distribution, day and time, BAC.	Drivers 21%, passengers 38%, pedestrians 41%, males 85%, males 20-39 years 66%. Injuries: brain 25%, spinal cord 6%, multiple sites 54%. Driver fatality per 10,000 vehicles: cars (4.6), motorcycle (21.3). Pedestrian 20% aged <10 years, passengers 25% fell/jumped from moving vehicle. Alcohol associated with 76% of those aged >10 years. BAC > 80 mg/dl observed in 1/3 drivers and 69% male pedestrians, BAC > 120 mg/dl 62% fatalities. 46% of fatalities occurred on Friday and Saturday	Method to identify eligible cases not stated. Measurement bias reported for incomplete or delayed blood alcohol testing.
2. Sinha et al [24]. (1981), PNG, Case series Retrospective	305 trauma-related forensic autopsies, (171 RTI-related) PMGH, 1976-80.	Road user type, age, sex, injury distribution, day and time, risk factors, BAC.	Drivers 17%, passengers 46%, pedestrians 36%, males 86%, 82% < 35 years. Injuries: head 65%, chest 51%, abdomen 37%, spine 18%, fracture skull 57%, brain injury 53%, and fracture ribs 39%. Chest injuries higher in drivers (65%), head injuries higher in passengers (71%). Risk factors: driver lost control 60%, passengers fell off a truck 60%. Alcohol associated with 85% of drivers and 90% of adult pedestrians. 53% BAC >80 mg/dl. 57% occurred over weekend, 44% occurred between 6 pm and 6 am. 66% died at the crash site or soon after.	Post mortem records supplemented with hospital admission notes. Incomplete records excluded, measurement bias reported for incomplete or delayed blood alcohol testing. Comparison between RTI and non-RTI related deaths limited to those with spleen injury.
3. Palmer [25] (1982), PNG, Case series Retrospective	353 forensic autopsies (97 RTI-related) Goroka hospital, 1978-82.	Injury cause, distribution	Dead on arrival to hospital 81% (n = 79), head injuries 71% (n = 56), most deaths followed ejection or jumping from a moving vehicle. Of those who died during hospital admission, 61% had head injuries.	Method to identify eligible cases not stated
4. Gee et al [26]. (1982), PNG, Case series Retrospective	36 trauma-related spinal cord injury admissions PMGH, Lae, and Madang hospitals,1978-81.	Age, sex.	RTI-related spinal cord injuries 34% (n = 12). For all trauma related spinal cord injuries: 88% Male, mean age 26 years	Incomplete information in case records reported. Selection bias not reported.
5. Lourie et al [27]. (1983), PNG, Case series Prospective	209 RTI-related admissions A&E PMGH, 1982-83.	Road user and vehicle type, passenger seating, seatbelt use	Drivers 15%, passengers 72%, pedestrians 13%, males 82%, those aged > 16 years 87%, 3 dead on arrival, 22% admitted, 40% drivers/front seat occupants injured, 13% front-seat occupants wore seatbelt. 28% passengers on the back of utility trucks, and all injured. Vehicles: cars 33%, utility trucks 27%. 1/3 vehicles no seat belts fitted.	Presence of investigator blinding of outcome not stated. Limitations of study such as selection and recall bias, well described, Measurement bias for alcohol association reported. While risk factors for RTI identified, study design precluded quantification of risk.
6. Sinha et al [28]. (1989), PNG, Case series Retrospective	363 RTI-related forensic autopsies PMGH, 1976-85. Total trauma-related autopsies 608, 60% RTI related.	Road user type, age, sex, BAC, injury distribution, day and time, if death occurred before arrival to hospital.	Drivers18%, passengers 45%, pedestrians 34%, males 83%, 15-44 years70% Injuries to the head 43%, chest 30%, abdomen 8%, spine and pelvis 10%, fracture skull 15%, brain injury 17%, intracranial bleed 10%, fracture ribs14%. Raised BAC (> 80 mg/dl) in 48% of drivers tested (n = 16/33), and 67% of pedestrians (n = 20/30) 54% occurred over the weekend, 40% 6 pm to 6 am 2/3 died at the crash site or soon after	Reports on five year extension to earlier study by Sinha et al [28].; Measurement bias reported for incomplete or delayed blood alcohol testing.
7. Cooke et al [29]. (1992), PNG, Case series Retrospective	573 RTI-related forensic autopsies PMGH, 1962-89 Total autopsies 1279, 45% RTI-related	Road user type, sex.	75% males (n = 432). Total males: drivers 12%, passengers 35%, pedestrians 28% Annual crude data shows increasing RTI-related fatality trends over 4 decades	Limited reporting on methodology. Bias due to incomplete datasets, identified.
8. Posanau [30] (1994), PNG, Case series Prospective	188 RTI-related admissions A&E PMGH, 1990. Total road traffic crashes 104.	Road user type, age, sex, time and day of crash, association with alcohol.	74% males (n = 136), 48% aged 18-29 years. 49% of road traffic crashes associated with alcohol. Most crashes occur in first part of day, but for alcohol related crashes, more common at night and early morning. Also alcohol related crashes more common on weekends. Road users – 25% drivers, 61% passengers. For those admitted to hospital, 43% drivers, 54% of passengers and 31% of pedestrians.	Data source; administered questionnaire, clinical assessment, and autopsy reports. Validation of definition of alcohol-related road traffic crash and assessment of alcohol intoxication not stated. Presence of investigator blinding of outcome not stated.
9. Maharaj [31] (1996), Fiji, Case series Retrospective	140 spinal cord injury admissions Fiji medical rehabilitation unit, 1985-94.	Sex, distribution of injuries.	Trauma-related spinal cord injuries 54% (n = 75), 25% RTI related (n = 19) Fijian males comprised 87% of trauma-related spinal cord injuries.	Included both trauma and non-trauma-related spinal cord injuries. Results reported as event counts and proportions.
10. Mathew et al [32]. (1996), PNG, Case series Retrospective	454 trauma-related admissions Mendi hospital, 1993.	Injury distribution, length of hospital stay.	RTI related trauma admissions 14% (n = 63) Injuries to head 37% with 2 deaths, chest 14%, abdomen 6%, fractures and dislocations 29%, Average length of stay for RTI, 9.6 days, median 5 days, range 1-51 days, same for assaults	Methods of data collection not reported. Exclusion of 15% of patient charts due to incomplete data.
11. Liko et al [33]. (1996), PNG, Case series Prospective (*x*2) Retrospective (x1)	274 head injury admissions PMGH and Goroka hospital, 1984-93.	Injury distribution.	RTI related head injuries 49% (n = 134). Distribution of admissions, 55% to PMGH and 45% to Goroka hospital Head injury case fatality 21%	Method to identify eligible cases not reported. Selection bias not reported.
12. Watters et al [34]. (1996), PNG, Case series (x4) Prospective (*x*2) Retrospective (x1) Prospective and Retrospective (x1)	667 trauma-related admissions A&E, PMGH, (35 days over 3 months); 154 trauma admissions PMGH general surgical unit (over 1 year); 88 trauma-related deaths prior to admission to PMGH(8 months); 50 deaths during admission (over 18 months) PMGH, 1992-93	Injury cause, distribution, length of hospital stay.	RTI A&E admissions 6%, hospital admissions 30% Mean stay for RTI, 26 days, twice as long compared to assault, accidents and sports Deaths prior to admission RTI 30% (n = 26), assault 45% (n = 40). Total head injuries 37% (n = 33), RTI related n = 24.	Four studies over a period of time. Recruitment dates for trauma-related admissions randomised. Method to identify eligible cases as well as selection bias not reported.
13. Ravia [35] (1999), Yap, Case series Retrospective	100 injury-related admissions A&E Yap State Hospital,1996-98	Road user type, association with alcohol, residence	Drivers 12, passengers 2, pedestrians 0, RTI-related injury admissions 14% (n = 14). Five times increase in RTI among drivers from n = 2 to n = 10. 88% of all injuries associated with alcohol	Methodology section limited. Biases not reported.
14. Ponifasio [36] (2001), PNG, Case series Prospective	213 abdominal trauma related admissions to PMGH (Feb 1992- Jun 1994), and 98 to Lae hospital,1996	Distribution of injuries	RTI-related non-fatal abdominal injuries 10% (n = 30), 311 admissions: 214 males aged 13-56 years, mean 22 years.	Method of data collection and biases not reported.
15. Prasad et al [37]. (1981), Fiji Case series – hospital and population-based Retrospective	2277 road traffic crashes Fiji, 1980; 872 road traffic crashes Western division province Fiji, 1980; 165 RTI-related admissions Lautoka hospital, 1980.	Road user type, age, sex, ethnic group, injury distribution, day and time, risk factors.	Road traffic crashes: Fiji 1%, WD 6%. WD 9% of road crash victims required hospital admission. Risk factors: dangerous driving, (66-70%); pedestrian fault (7.5%); alcohol (4%); speed (2.2-2.5%); mechanical defects. Most crashes occurred during the day and in dry weather conditions, 75-77% crashes occurred on tar-sealed roads. Hospital admissions: 73% male, 24% aged 20-29 years, 60% Fiji-Indian, 32% Fijian, 40-46% head and neck injuries. Crashes increased during school holidays, over weekend, afternoons and evenings. Children more likely to be injured as pedestrians. Falling off the back of utility vans common.	Limited reporting on methodology and quality of data. Risk factors indicated, but study design precluded quantification of risk. No biases reported.
16. Jayasuria [38] (1991), PNG, Ecological study Retrospective	3202 motor vehicle crash-related injury registrations (314 deaths), Royal PNG Constabulary database for traffic accidents, 1987; National statistical office data for motor vehicle crash-related injury trends prior to 1980. Motor vehicle registry, and Licensed drivers National statistical office	Motorisation, crashes, injuries/deaths by road user and vehicle type, age, time of crash	Injuries and deaths: drivers 18%, passengers 61%, pedestrians 19%, 23% 26-30 years. Deaths: Drivers 13%, passengers 51%, pedestrians 34%. Trends in injuries and deaths by road user type have not changed much since 1970. However, for pedestrians the fatality index (deaths/deaths and injuries) there was a significant increase from 11% to 18% for the period 1970 to 1987. Rate of pedestrian injury per 100000 population = 31 (21-30 years) 20 (0-10 years), and 18 (30-50 years). Total deaths and injuries by vehicle type: 44% utility - small pick-up trucks), 16% heavy goods vehicles - single unit trucks, 18% pedestrians, 10% cars. Pedestrians, 32% aged < 10 years. Crash rates by vehicle type (1982-87), declining, but buses increasing, to four times the rate of cars and 2.4 times that of utility vehicles. Severity of crash and vehicle type (1987); highest rates of injury and fatality: drivers (motorcycles), passengers (utility, heavy goods vehicles, buses). Increased crashes from 6 pm, and over weekends (Friday and Saturday)	Data collection, exclusion criteria, not reported. Results reported as trends in unadjusted event counts, proportions, and rates.
17. Hills et al [39]. (1993), PNG, Ecological study Retrospective	4485 motor vehicle crashes-related injury registrations, Royal PNG Constabulary database for traffic accidents, 1991. Other LMIC countries: Bandung n = 1059, (1990) Malaysia n = 37955, (1992) Karachi n = 1261 (1991) Colombo n = 946 (1991-92) Total crashes 45702.	Road user type, age, vehicle type, collision characteristics, day and time of crash.	Pedestrians ranked 1st or 2nd for RTIs Peak age for RTIs: 21-30 years (PNG, Karachi, Colombo); 16-25 years (Malaysia, Bandung) Pedestrians: 6-10 years peak for PNG and Malaysia. Vehicle type: utility trucks - PNG 45%, other LMIC countries – mostly motorcycles. Vehicle collisions: PNG – rollover, Malaysia side or 90°, Bandung collisions with pedestrians. Number of vehicles in collision: PNG > 70% single vehicle. Most injuries occurred in rural areas (70%), associated with higher speed, and delayed medical aid. Common day and time for RTIs, PNG - Friday and Saturday, usually alcohol-related; between 8 am to 8 pm.	Methodology for data collection not reported. Results reported as unadjusted proportions comparing countries. Identified variation in defining injury severity.
18. Nelson et al [40]. (1991), PNG, Ecological study Retrospective	5772 motor vehicle crashes-related injury registrations (1921 deaths), Royal PNG Constabulary database for traffic accidents, 1984; Motor vehicle registry, and Licensed drivers National statistical office, 1980-84; Traffic survey 1984 on vehicle occupancy.	Road user type, Vehicle type, ownership and occupancy characteristics Crash severity and crash rates	Fatalities: drivers 17%, passengers 46%, pedestrians 36%. Utility responsible for 38% of crashes, 65% passenger fatalities. 34% crashes involve single vehicles, RR of fatality; Driver - utility 0.99, bus 0.88, car 1.57; Passenger: utility 3.1, bus 2.4, car 1.3. RR of crash involvement; utility 1.07, bus 2.14, car 0.87. Open back vehicles responsible for high number of fatalities per crash, occupants thrown out of vehicle. Potential 27% reduction in crash fatalities if utility and heavy goods vehicles restricted to designed passenger occupancy	Methodology for data collection, exclusion criteria, not reported. Results reported as trends in unadjusted event counts, proportions, and rates, for motorisation, crashes, deaths and injuries stratified by road user, vehicle type, age and time of crash. Reported limitations to notification, collection, assumptions, and modelling – eg multiple vehicle crashes reported as total injuries per event therefore unable to identify vehicle with casualties. Effect estimates identify RR for drivers and passengers based on type of vehicle.
19. Hills et al [41]. 1996, PNG, Case control	893 drivers undertaking roadside breath alcohol testing. 12 road sites, two different sites per night, 10 pm - 2 am, Thursday to Sunday, for 5 weeks (1990).	Sex, ethnic group, BAC	97% males, 98% PNG Nationals, BAC > 80 mg/dl 24% Males, 21% Females, 24% drivers. (Driver BAC > 150 mg/dl 12%, BAC > 215 mg/dl 4%). Friday night early Saturday morning 29% drivers BAC > 80 mg/dl, Thursday 27%, Saturday 25%, Sunday 8%. BAC > 80 mg/dl 17% after 22 cases excluded. BAC > 80 mg/dl PNG nationals (citizens) 22% Non-PNG nationals 33% sampling error significant (p < .05)	Non-randomised sampling by site, day, time. No response rate provided, nor vehicle type. 22 participants excluded due to test conducted < 20 minutes from the last drink. Measurement bias for BAC not reported.
	37 RTI-related admissions A&E, PMGH undertaking BAC tests, 11 pm to 3 am, for 15 weekends (1990).	Road user type, BAC and breath alcohol concentration.	Drivers 27%, passengers 62%, pedestrians 11%. 2 fatalities, 2% hospitalised, 76% discharged. BAC > 80 mg/dl in 50% of A&E admissions, and 8 drivers (n = 8)	Little information on methodology and distribution of blood and breath alcohol concentration results among cases. Results reported as events with no adjustment for confounding and bias, including differences in testing methods.

## Results

Nineteen studies [23-41] published between 1980 and 2010 met the inclusion criteria with the most recent RTI-specific study published in 1996 [41]. One study was published in two journals [41,42], so the study with less information was excluded [42]. Fifteen studies were case series; including ten studies identifying cases from retrospective records [23-26,28,29,31,32,35,37], three studies from prospective records [27,30,36], and two studies from both retrospective and prospective records [33,34]. There were three ecological studies [38-40], and one study was a case control study [41]. All but three studies were undertaken in Papua New Guinea (PNG), the exceptions being retrospective case series conducted in Yap [35], and the Republic of Fiji (Fiji) [31,37]. Eleven studies focused on describing the overall burden of RTIs and type of road users injured [23-25,27-29,37-41], eight studies highlighted the contribution of RTIs to trauma-related injuries [26,31-36], and 11 studies described the anatomical distribution of injuries sustained in a road crash [23-26,28,31-34,36,37]. While 11 studies provided information on exposures such as alcohol, seatbelt use and vehicle type [23-25,27,28,30,37-41], only two studies, published in 1991 and 1996 [40,41], incorporated epidemiological designs that enabled identification of aetiological factors for RTIs.

### Appraisal of study methods and quality

Although the majority of studies were case series, there was wide variation in study design and quality. Most of the studies conducted outside PNG were small except for one study in Fiji reviewing national road crash data (n = 2277) [37]. In general, PNG studies involved larger study samples (up to 5772) including three ecological studies drawing on the national road crash database [38-40].

The methodological quality of the studies or the ability to appraise this was compromised by inadequate reporting of population characteristics, case definitions, recruitment methodology (source, eligible and study population), exclusion criteria, data collection and analysis, response rates, and identification of potential biases and confounders. Most research designs failed to consider possible biases in analysis and the interpretation of findings. Since many studies reviewed post mortem records, potential cases may have been excluded if they had not presented to the hospital setting. The majority of studies were retrospective, and the quality of data sources, (e.g. completeness, accuracy) was unspecified. This makes it difficult to ascertain the extent to which misclassification and underreporting may have been a source of information and recall bias. No studies reported whether outcome measures or blood alcohol concentration (BAC) measurements were assessed blind to exposure information, nor whether measurement bias was considered when estimating alcohol concentration from breath or blood samples.

Regarding measures of occurrence and effect, most of the studies reported unadjusted event counts and proportions, with only three studies providing population-based rates [23,38,40], and an ecological study reporting effect estimates [40].

Acknowledging these limitations, Table 1 summarises studies investigating the burden and aetiological factors for RTIs in Pacific countries, all of which were published more than a decade ago. The key aspects of these are described next.

### RTI-related fatalities in PNG

In a study of 1,279 post mortems from all causes of deaths in all ages conducted at Port Morseby General Hospital (PMGH) for the years 1962 to 1989, RTIs (n = 573) accounted for 45% of all deaths, 75% of whom were male [29]. Another post mortem study focusing only on trauma-related fatalities in all ages (n = 608) at PMGH (1976 to 1985), showed that RTIs were responsible for 60% of these deaths, 83% of whom were male [28]. Notable findings in other studies included the high proportion of road deaths that occurred at the crash site or before arrival to PMGH (66%) [24] and Goroka Hospital (81%) [25], as well as the disproportionately high number of fatal crashes on weekends and during early morning hours [23,28].

Motor vehicle passengers (46–51%) and pedestrians (34–36%) were the road user groups commonly involved in fatal crashes [38-40]. Head injuries were reported as the commonest cause of RTI related deaths, with in-country differences observed between Port Moresby (43%) [28] and Goroka (71%) [25]. In a study of 305 trauma related post mortems undertaken in PMGH, chest injuries were found to be more common among drivers (65%) whereas in passengers, head injuries were more common (71%) [24].

### RTI-related hospital admissions in PNG

Studies suggest RTIs were responsible for 14–30% of trauma admissions [32,34], 49% of head injury admissions [33], 10% of abdominal trauma admissions [36], and 34% of trauma-related spinal cord injury hospital admissions [26]. The average length of stay was usually longer for RTIs compared to other trauma-related injuries with variations between hospitals (Mendi = 10 days, PMGH = 26 days) [32,34].

### RTI-related studies in pacific countries other than PNG

RTIs contributed to 14% of injury related admissions (n = 100) to the accident and emergency (A&E) department in Yap hospital [35], and 25% of trauma-related spinal cord injury admissions (n = 75) to a rehabilitation unit in Fiji [31]. A Fiji study examining national (n = 2277) and Western Division) Province (n = 872) data reported deaths in 1% and 6% of crashes respectively [37]. In this study, 40% of those hospitalised had sustained head and neck injuries, while injured children were more likely to be pedestrians [37].

### Potential contributors to RTIs

Although we did not identify any aetiological studies with appropriate control (unexposed) groups, several studies suggested factors that were likely to be important contributory factors to road crashes.

An ecological study drew attention to vehicle type, noting increased risks of crashes involving buses and open-back utility vehicles compared with cars [40]. Several other studies also noted the high proportion of utility vehicles involved in crashes [27,38,39]. When combined with road user type, passenger fatalities were noted to be highest for utility vehicles and lower for buses and cars [40]. Between 25 and 60% of passenger deaths were due to falls from utility vehicles [23,25], attributed largely to vehicle overcrowding [40].

In contrast to passenger injuries, driver injuries were reported to be highest for cars, with some studies attributing up to 70% of RTI fatalities to driver error [24,37]. One review of 121 post mortem records in PNG identified that motorcycle drivers were five times more likely to die in a road crash than car drivers [23].

Several studies commented on the role of alcohol in RTIs. In a roadside survey of motor vehicle drivers (n = 893), and review of RTI related drivers assessed at the PMGH emergency department (n = 10), BAC levels above 80 mg/dL were detected in 24% and 80% of drivers, respectively [41]. Post mortem data on trauma-related cases revealed BAC levels greater than 80 mg/dl in 48% of drivers and 67% of pedestrians [28]. In 188 RTI-related patients involved in 109 crashes, 49% of crashes were associated with alcohol [30]. Several studies suggested that alcohol-related crashes were more common at night, early morning, and during weekends [23,28,30,41]. While not designed as a formal aetiological investigation, one study in Fiji attributed 4% of road crashes to alcohol and 2.5% to speed [37].

## Discussion

The literature regarding the burden and risk factors in the Pacific region remains largely limited to descriptive studies in PNG with important limitations in their ability to examine the population-based burden of road crashes and investigate related risk factors. No RTI focused epidemiological studies have been published for over 15 years and few were conducted outside PNG. Notwithstanding these limitations, the published literature indicates that road crashes are a major public health problem. Studies from PNG reported that RTIs contributed to at least 40% of all deaths, 60% of trauma-related deaths, one third of trauma-related hospitalisations, and 40% of all head injuries. Most (two thirds) RTI-related deaths occurred prior to hospitalisation with related fatalities particularly high among pedestrians and passengers. Although head injuries were the most commonly reported RTI-related injury, chest injuries were reported to be particularly common among motor vehicle drivers. The available literature suggests that vehicle type (open-back utility), utility vehicle overloading, and alcohol are potentially important risk factors for RTI.

This review was limited to scientific publications and the English language. Therefore, it excluded the grey literature and routinely collected data such as police annual reports and national hospital and mortality statistics, most of which report events but provide little critique or analysis. The diverse methodological characteristics of the studies identified made it inappropriate to undertake a metaanalysis, or infer that the findings from these largely PNG focused studies can be generalised to Pacific countries more generally.

Nevertheless, this review adds to the evidence base of previous reviews on RTI in less resourced settings [7,43,44], focusing in this case, on Pacific countries which are often absent or overwhelmed by statistics from countries with very large populations in the Western Pacific region. A defining feature of this review is the paucity of robust and contemporary epidemiological RTI studies from Pacific countries, suggesting research attention to RTIs in the region have not been a priority [45,46] and long overdue. However, the evidence available indicates that, as in other less resourced settings, RTIs contribute significantly to the burden of RTIs in Pacific countries and poor data should not delay action [47],

The review particularly reveals the need to undertake well-designed aetiological studies that can quantify the contribution of important context-specific risk factors associated with serious road crashes. Indicative findings from the review suggest a key area requiring focused attention in PNG is improved safety considerations relating to the use of utility type vehicles [48]. While the approaches to address this must take into account the socio-economic and transportation implications for the communities involved [49,50], it has been argued that restricting passenger numbers through vehicle occupancy capacity limits may have the potential to reduce crash fatalities by almost 30% [40]. Other areas amenable to well-implemented legislative interventions in the region include the use of seatbelts [51] and deterrents to drink driving [16]. Given the high proportion of road deaths at the crash scene, greater attention is required to health system improvements including pre-hospital care and the training of first responders [2].

The World Report on road traffic injury prevention identifies the need for a comprehensive multi-sectoral, integrated, systems approach that focuses on improved information systems, institutional capacity strengthening, research that quantifies common modifiable risks, and resources for targeting these [2]. Reliable and sustainable injury data surveillance [52-55] including secondary data routinely collected from hospital and police records, comprises an important foundation for monitoring and evaluating road safety strategies in the Pacific context [19,39]. This would be strengthened by the standardisation of RTI case definitions, data collection processes, analysis and reporting from surveillance systems [56], while ensuring dissemination approaches link findings to interventions, policies, context-specific research and funding mechanisms. As noted elsewhere [57], a critical requirement for identifying and implementing effective road safety strategies is a skilled workforce integrated across relevant stakeholder agencies including legislation, economic policy, capital infrastructure, road engineering, vehicle design, and health care.

## Conclusions

This review found that the burden of road traffic injuries in PNG and other less resourced Pacific countries is significant, however, the contribution of modifiable risks for RTI remain poorly quantified. While some studies revealed the likely importance of factors such as alcohol misuse and vehicle type, effective public health and road safety policy requires attention to population-based studies that can identify and quantify the contributors to RTI in the Pacific context. Improving the quality of secondary data sources from routinely collected hospital and police surveillance systems is essential.

## Abbreviations

A&E: Accident and emergency; BAC: Blood alcohol concentration; PNG: Papua New Guinea; PMGH: Port Moresby General Hospital; RTI: Road traffic injuries.

## Competing interests

The authors declare that they have no competing interests.

## Authors’ contributions

JH conducted the literature search, selected included papers, critically appraised the papers, developed the evidence tables, and wrote the initial draft of the paper. SA and RJ contributed substantially to the design of the evidence tables, the outline of the paper, interpretation of findings and writing the paper. All authors read and approved the final manuscript.

## Pre-publication history

The pre-publication history for this paper can be accessed here:

http://www.biomedcentral.com/1471-2458/12/479/prepub

## Supplementary Material

Additional file 1 Search string applied for the review. Click here for file

Additional file 2 **PRISMA checklist for systematic reviews[**[58]**].** Click here for file
