# Supplementary material for: Burden of road traffic injuries and related risk factors in low and middle-income Pacific Island countries and territories: a systematic review of the scientific literature (TRIP 5)
Source: BMC Public Health. 2012 Jun 25;12:479. doi: 10.1186/1471-2458-12-479 (PMC3490885; doi:10.1186/1471-2458-12-479)
Supplement: Additional file 1 — Search string applied for the review. [file 1471-2458-12-479-S1.docx]

The following electronic search string was developed for Medline noting that for Population group, all Pacific countries and populations were included in the initial search:

1. Population: (Guam$ or Trust Territor$ or Northern Mariana$ or Palau$ or Caroline island$ or Marshall Island$ or Yap$ or Federated States of Micronesia$ or FSM or Micronesia$ or Nauru$ or Kiribati$ or Gilbert island$ or Papua New Guinea$ or Bismarck Archipelago$ or Solomon Island$ or Vanuatu$ or New Caledonia$ or Melanesia$ or Fiji$ or Fiji island$ or Rotuma$ or American Samoa$ or Western Samoa$ or Samoa$ or Tuvalu$ or Tokelau$ or Niue$ or Cook Island$ or Tonga$ or Tuamotu$ or Pitcairn$ or Norfolk$ or Wallis$ or Futuna$ or Rapa Nui$ or Easter island$ or French Polynesia$ or Tahiti$ or Marquesas Island$ or Society island$ or Savai$ or Polynesia$ or Pacific or Oceani$).mp.
2. Exposures: (alcohol or wine or spirit or beer or seatbelt or helmet or head protective device or helmet or sleep$ or fatigue or apnoea or shift work or snor$ or conspicuity or visibility or illumination or visual or headlight$ or light or traffic or colour or color or contrast or road or dirt or gravel or tar seal or weather or rain or wet or vehicle or car or motor or cycle or pillion or automobile or bike or moped or motorbike or motorvehicle or motorcar or walking).mp.
3. Outcome: ((road or traffic or accident or crash or collision) and (injur$ or disab$ or hospital$ or wound or morbid$ or prognos$ or mortalit$ or death$ or health status or fatal$)).mp.
4. Incidence studies
   1. exp Mortality/
   2. exp Follow-Up Studies/
   3. mortality (sh)
   4. Predict: (tw)
   5. Prognosis: (tw)
   6. course (tw)
   7. or/1-6
5. Aetiological studies:
   1. exp cohort studies/
   2. exp risk/
   3. (relative and risk) (tw)
   4. odds.mp. and ratio: (tw) [mp=protocol supplementary concept, rare disease supplementary concept, title, original title, abstract, name of substance word, subject heading word, unique identifier]
   5. case.mp. and control: (tw) [mp=protocol supplementary concept, rare disease supplementary concept, title, original title, abstract, name of substance word, subject heading word, unique identifier]
   6. or/1-6
